# Effects of magnification modes and location cues on visual inspection performance

**DOI:** 10.1371/journal.pone.0213805

**Published:** 2019-03-14

**Authors:** Fion Choi Hung Lee, Siu Shing Man, Alan Hoi Shou Chan

**Affiliations:** 1 Division of Business, UOW College Hong Kong/Community College of City University, Hong Kong, China; 2 Department of Systems Engineering and Engineering Management, City University of Hong Kong, Hong Kong, China; University of Muenster, GERMANY

## Abstract

Image magnification often results in disorientation through loss of orientation and location during inspection. This study investigated the effects of three different magnification modes viz. full screen, circular, and fixed-area on visual inspection performance. Also, to improve participants’ global orientation with respect to the original product, location cues in the form of halftone landmarks were introduced as a job aid and their effectiveness on inspection performance was examined. Twenty-eight undergraduates participated in the experiment. Significant magnification mode effect was found, but the location cue effect was found non-significant. The results suggested that the presentation of content/contextual information on one single screen should be considered together with the nature of the visual task and participants’ search behaviors, and that the aid of location cues might be useful when the visual task demanded a high level of search memory and/or an unsystematic search strategy was employed by inspectors.

## Introduction

Visual inspection is one of the quality control processes in manufacturing industry and plays an important role in our daily live [1–4]. Previous studies have focused on obtaining an in-depth understanding of and improving human visual related task performance from a diversity of perspectives. For instance, Yu and Yang [5] conducted a study to examine the age-related changes in visual lobe shape characteristics and its relationships to visual search performance. Liu and Yu [6] used an eye-tracking technique to investigate the influence of social presence on eye movements in visual search tasks and found that the presence of an audience could evoke a social facilitation effect on response time in visual search tasks. Wahn, Schwandt [7] found that the performance of joint visual search can be improved by employing a tactile or an auditory display to exchange gaze information about the search partner.

With miniaturization of electronic and mechanical components of consumer products, video magnifiers have been designed for human visual inspection. The product to be inspected is placed under a camera and the magnified image is presented on a display. In contrast to hand-held magnifiers and microscopes, the video magnifier systems show the benefits of reducing bending postures over eyepieces and back fatigue in prolonged working with microscopes [8]. Besides, video magnifiers that connect to computer systems help keep track records of inspected product images and offer some advanced features like color inversion, smoothing, and multiple views. Nevertheless, this artificial technique of image enlargement displays only a small portion of the product on the screen at a time. Although the magnified window can be moved around by inspectors to enable larger coverage of a product to be inspected, it is easy to lose track of the present location and orientation within the sequence of complex scenes on the product surfaces. Apparently, the problem of disorientation and hence the consideration of screen space allocation for the original product image and magnified image are important issues for the human computer interface designers.

Studies on the interface design of video magnifiers are limited. A study conducted by Zhao, Rau [9] suggested that a video magnifier equipped with auditory output support and yellow-highlighted background could help improve the search performance of the elderly users. It could be due to the larger eccentricity effects on latencies and errors exhibited in older adults than in younger adults [10] and thus supplementary tools may be useful. In some situations where multiprocess-handling workers cannot use their hands because of efficiency or hygiene reasons, Kotani, Nakajima [11] proposed a hands-free visual inspection system using saccadic latency, a temporal characteristic of saccadic eye movements, and the result of the average defect detection rates was promisingly high at 99.4%. Lin, Chen [12] investigated the use of scanning electron microscope in semiconductor industry and the study revealed that increasing the luminance contrast between target and background and the size of the LCD monitor could reduce eye fatigue and improve inspection performance. More recent studies on relationships of inspection performance on visual displays with human visual ability in terms of visual lobe shape were reported for different display movement velocity [13] and inspectors of different ages [5].

**Fig 1:**
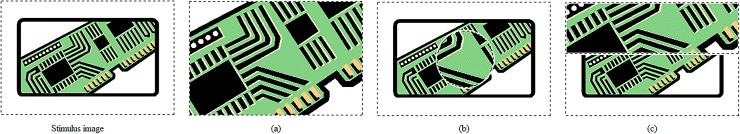
An illustration of stimulus images for different magnification modes. (A) full screen, (B) circular, and (C) fixed-area.

A more comprehensive review of user interfaces on video magnifiers shows that there are different magnification modes available for use in computer systems [14]; however, there was also a suggestion that the added variety of magnification modes in the new generation of video magnifiers might not necessarily lead to more effective systems. One common mode of magnification is the full screen magnification in which the entire screen is taken over by the magnified image of a part of the original product (Fig 1A). Inspectors’ eye fixations can easily move around on the magnified image in this mode because there is only one area under consideration. However, it has the disadvantage of causing a loss of orientation in participants during navigation as it uses the full screen area for the content information for only the part of the product under magnification. Inspectors can view the magnified part of the product image at any one time, but cannot link it easily to the global context of the original product image. Other common modes of magnifying objects are the circular mode (Fig 1B) and fixed-area mode (Fig 1C) in which the magnified image occupies a certain proportion of the screen and the remaining screen space displays part of the original product image. The window for magnified content information is usually smaller in comparison to the full screen magnification in order to provide global contextual information on the screen. In the circular mode, inspectors can know where the focus area (underneath the lens) is located in relation to the rest of the product image. However, they usually face the difficulty and challenge of mentally integrating the global contextual and local content information. In the fixed-area mode, the magnified image is always shown at the top of the screen while part of the original product image is shown on the rest of the screen underneath. The differences among the modes are the type of shape and the area of magnification. The objects in the magnified window are updated as the cursor moves on the screen. Because the contextual and content information is consistently located in two pre-designated areas, there is less difficulty for inspectors in integrating the two types of information. However, compared with the in situ magnification in the circular mode, the problem of abrupt transitions on two perceptual levels required from the magnified image window and the original product window may not be so good for inspection performance. Hence, special care needs to be paid to the interface design so that sufficient space is allocated for presenting the contextual and the content information on one single screen.

In order to better understand the usefulness of content and contextual information in general fault-searching tasks, this study examined inspection performance of participants under the three basic modes of magnification, viz. full screen, circular, and fixed-area modes, with a simulated visual inspection task. Based on the anticipated problems and the intrinsic benefits of showing content and contextual information on the screen, it was believed that participants’ visual inspection performance would differ under different modes of magnification. If the content information was more important than the contextual information in the task employed, full screen mode would show the best inspection performance. In contrast, if the contextual information was more essential to the task, full screen mode would then be the worst among the three magnification modes.

Kristjánsson [15] revealed strong evidence that memory for locations played an important role in a visual search task. It has also been suggested that global visual contextual information guided the deployment of visual attention and constrained where to look, so as to facilitate search for and recognition of objects embedded in complex displays [16]. In this study, with regard to the disorientation problem mentioned above, half-toned location cues (+, ↑, ↓, ←, → ↗, ↖, ↙, and ↘) were used and embedded in the stimulus image with the objective of improving the inspectors’ sense of global orientation of the product image. The location cues may serve as landmarks and help inspectors to memorize the visited locations and ultimately improve their search performance. Given the potential usefulness of location cues, the effects of four levels of location cues, presented in the form of artificial landmarks and superimposed on specific locations on product image, were examined in this study. The higher the level of cues presented, the greater the number of location landmarks were placed on the product image. It was hypothesized that with the use of location cues, the sense of global context of the product image and thus the visual search performance would be improved. Higher level location cues were expected to result in greater improvement than lower level ones.

To evaluate the effectiveness of different magnification modes and the effects of different levels of location cues on visual inspection performance, the objective performance measures of speed and accuracy were analyzed together with the subjective evaluation of NASA Task Load Index (NASA-TLX) paradigm [17] which was administered electronically. This evaluation paradigm measured participants’ mental demand, physical demand, temporal demand, own performance, effort, and frustration, and was used to gain some understanding of participants’ perceptions of different magnification modes and location cue conditions.

## Materials and methods

### Design

The effects of magnification modes and location cues were tested under two magnification powers of 2X and 3X. It is known that the number of objects presented on the magnified window decreases with increasing magnification level, and thus increasing the number of eye fixations and the overall inspection time within the search area. Stimulus objects magnified at 2X or 3X in the present study were discernible for making accurate decision. In order to reduce the total test duration for each participant, a mixture design experiment was used. The magnification mode (full screen, circular and fixed-area) was a within-subject factor whereas the levels of location cues (no cue, level 1, level 2 and level 3) and magnification powers (2X and 3X) were between-subject factors. Each participant was tested with two levels of location cues either no cue and level 2 or level 1 and level 3. Hence, six test conditions (3 magnification modes × 2 levels of location cues × 1 magnification power) were given to each participant. Table 1 shows the six test conditions in the four test blocks. Participants were randomly assigned to one of the four blocks and the sequences of the six test conditions within each block were counterbalanced across participants for reducing order effect. In each test condition, there were, one target-present practice trial and four testing trials in which two were target-absent. Participants were informed of the occurrence of target-absent trials but did not know the 50% target occurrence rate.

**Table 1:** Four blocks of test conditions.

No.	Block A	Block B	Block C	Block D
**1**	Full screen, No cue, 2X	Full screen, No cue, 3X	Full screen, Level 1, 2X	Full screen, Level 1, 3X
**2**	Full screen, Level 2, 2X	Full screen, Level 2, 3X	Full screen, Level 3, 2X	Full screen, Level 3, 3X
**3**	Circular, No cue, 2X	Circular, No cue, 3X	Circular, Level 1, 2X	Circular, Level 1, 3X
**4**	Circular, Level 2, 2X	Circular, Level 2, 3X	Circular, Level 3, 2X	Circular, Level 3, 3X
**5**	Fixed-area, No cue, 2X	Fixed-area, No cue, 3X	Fixed-area, Level 1, 2X	Fixed-area, Level 1, 3X
**6**	Fixed-area, Level 2, 2X	Fixed-area, Level 2, 3X	Fixed-area, Level 3, 2X	Fixed-area, Level 3, 3X

The sequences of the six test conditions (magnification mode, level of location cues, magnification power) within each block were counterbalanced across participants.

### Participants

Twenty-eight engineering undergraduate students (24 males and 4 females) of City University of Hong Kong with ages from 19 to 25 (median = 21) years participated in the experiment with course credit. All of them had binocular near acuity of at least nine (20/22 Snellen notation) on the Bausch and Lomb Orthorator and had previously participated in an experiment measuring visual lobe area and shape characteristics [18, 19]. The experiment was approved by the City University of Hong Kong’s Human Subjects Ethics Sub-Committee, and all subjects gave written informed consent before participation.

### Apparatus and software

A personal computer with a 2.8 GHz Pentium 4 processor and a 17″ LCD monitor (1280 × 1024 pixels) was used for stimulus presentation and response capture. The size of the viewing screen was 190 mm (horizontal dimension) x 130 mm (vertical dimension). Microsoft Visual C++ and Win32 Application Programming Interface were used to develop the virtual magnification interfaces and the simulated visual inspection task. The application programme was menu-driven and the experimenter could set a wide range of experimental conditions for testing various interface factors including magnification mode, level of location cues, and magnification power. A computer mouse was used for controlling stimuli, navigating, and inputting responses. During the experiment, the ambient illumination level was 400 lx. An adjustable chair was used for comfort and to ensure that the line of regard of participants was roughly perpendicular to and at the centre of the computer monitor.

### Stimulus image and viewing screen

In the simulated visual inspection task, the stimulus image was in the shape of square, which was fixed for all the three magnification modes, and had a size of 630 mm (horizontal dimension) × 630 mm (vertical dimension). In a target-present trial, the target object ‘X’ was positioned randomly amongst 1,500 non-target objects (‘A’, ‘K’, ‘M’, ‘N’, ‘V’, ‘W’, ‘Y’, ‘Z’) on the stimulus image. All visual objects were of 2.33 mm horizontally and vertically, subtending 16' of arc to participants’ eyes in both directions at a viewing distance of 500 mm. The minimum separation between the sides of adjacent objects was 16' of arc too. For reducing the effects of inter-object spacing and target surround density [20, 21], the target did not appear at the boundaries of the stimulus image and there were no more than four objects surrounding each target or non-target object.

**Fig 2:**
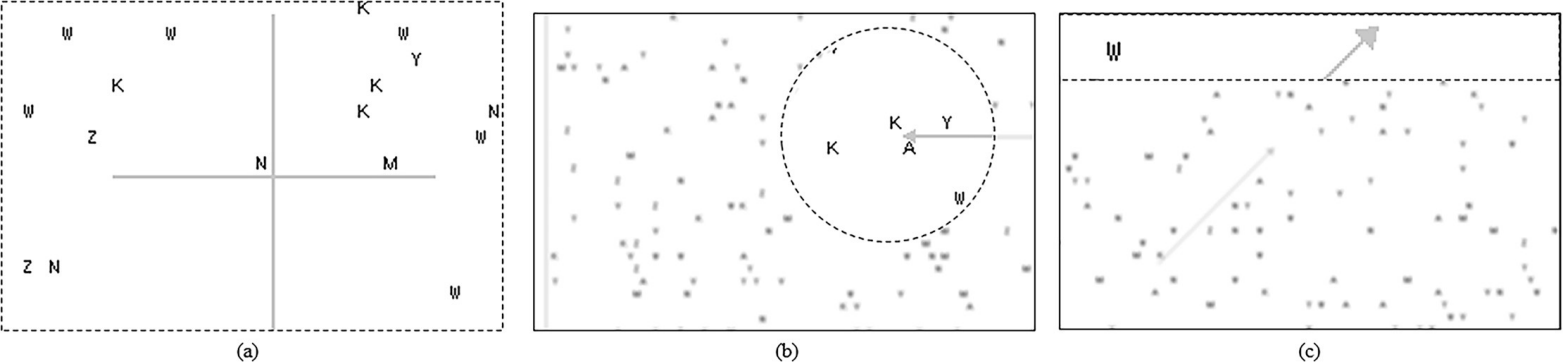
Examples of viewing screens. (A) full screen mode with a centre (+) location cue, (B) circular mode with a leftward arrow (←) location cue, and (C) fixed-area mode with a 45° inclined arrow (↗) location cue. The magnified windows are indicated by dotted lines.

The viewing screen (190 mm horizontal x 130 mm vertical) was set in positive polarity with dark objects (5 cd/m^2^) on a white background (150 cd/m^2^). Fig 2 shows the viewing screens for different magnification modes. The magnified image window (indicated by dotted lines), with the size and shape varied in accordance with the testing magnification mode, appeared on the viewing screen. It occupied the whole viewing screen in full screen mode and only a portion of the viewing screen in the circular and fixed-area modes. In circular mode, the circular image on the magnifying lens (radius = 39.65 mm) was updated as participants moved the lens. Similarly, in fixed-area mode, the image on the magnified image window (190 mm × 26 mm) was updated as participants moved the mouse cursor on the screen. The area of magnified window in the circular and fixed-area modes was the same so as to allow for meaningful comparisons. The content information was maximized in full screen mode whereas it was limited by the size of the magnified image window in the other two modes. However, in the circular and fixed-area modes, the showing of the blurred stimulus image on the viewing screen provided participants with contextual information whereas no such information was provided in the full screen mode.

In some testing trials, location cues were added with the intention of improving perception of global orientation with regard to the stimulus image. At level 1, there was only one location landmark (+) placed in the centre of the stimulus image. At level 2, in addition to the centre cue, four other location landmarks: ↑, ↓, ←, → were placed at the mid points of the top, bottom, left, and right imaginary meridians, respectively. At level 3, four more cues; ↗, ↖, ↙, ↘ were placed at the mid points of the 45°, 135°, 225°, and 315° diagonal meridians, respectively. To avoid any possible confusion with the stimulus objects, all the location cues were presented in halftones. All the images shown on the magnified window was magnified at 2X or 3X powers. The magnification referred to the ratio between the length of a magnified object on the magnified window and its true length. The magnification was symmetric in both meridians.

### Procedure

Participants were given verbal instructions about the objectives and procedures at the beginning of the experiment. They were then divided into four groups randomly and each group was tested with one of the four test blocks. Each participant received 30 inspection trials (6 test conditions x 5 trials). The first of five trials in each test condition was used for practice and was not included for analysis. There was no maximum allowable inspection time for each trial, but participants were instructed to search and make a decision as fast and accurately as they could.

Participants sat directly in front of a monitor at a viewing distance of 500 mm. The application software for generating the simulated inspection task was launched and participants were prompted to input their demographics data such as name, age, and gender. Each inspection trial started with the display of a cross in the centre of the screen. The cross comprised four (three grey and one black) bars extending from the centre to 0°, 90°, 180°, and 270° orientations and the black bar moved in a clockwise direction. Participants pressed the left button of the mouse when the bar appeared at the 90° orientation. A viewing screen then appeared and the search started at the centre of the stimulus image. Participants could move the mouse cursor to control the movement of the magnified window above the product image. Right after the detection of a target, participants pressed the right button to stop the motion of the magnifier on the screen, and made a left-click on the estimated target position appearing on the magnified window for confirmation. If no target could be found in a trial, they would press the ‘N’ key on the keyboard provided without right clicking the mouse. When a known mistaken input was made by the participant, a new trial of the same condition would be given for replacement and the mistaken input was discarded from later analysis.

Participant were required to fill out a computer-based Task Load Index (TLX) rating form after completion of each test condition and a weight form at the end of the experiment. A two-minute break was given to participants after each test condition. No feedback on response speed and accuracy was given to the participants. The whole test took about two hours to complete for each participant.

## Results

### Speed measure

The time elapsed from the onset of the stimulus image to the left-clicking on the perceived target position or the press of ‘N’ key to indicate target absence was taken as the inspection time (*IT*) for a trial. More specifically, the time to right click the mouse for making the claim of successful target location was the search time (*t*_*search*_), and the time to confirm the absence of target was the stopping time (*t*_*stopping*_) [22]. In this experiment, a total of 672 responses (28 participants × 6 test conditions × 4 testing trials) were collected for analysis. The overall mean and standard deviation (*SD*) for the *IT* data were 326 sec and 227 sec, respectively. Generally speaking, subjects made faster responses in target-present trials (mean *IT* = 260 sec; *SD* = 192 sec) than in target-absent trials (mean *IT* = 393 sec; *SD* = 240 sec). The mean and *SD* for the search time data of the hit responses were 239 sec and 184 sec, respectively. The relationship between the cumulative percentage of detection *P(t*_*search*_*)* and a certain search time *t*_*search*_ is usually expressed with a regression model [23, 24]. The search time data in this experiment fitted well to an exponential distribution and the search time model is shown in Eq (1) (*R*^*2*^ = 0.946).

$$\ln\left[1-P\left(t_{search}\right)\right]=0.29-0.0055t_{search}$$ (1)

The stopping time data was found to follow a lognormal distribution (goodness of fit statistic = 0.0534, *p* > 0.05). The mean stopping times for the misses and correct rejection responses were 287 sec (*SD* = 199 sec) and 393 sec (*SD* = 240 sec), respectively, while the overall average stopping time was 361 sec (*SD* = 234 sec).

**Fig 3:**
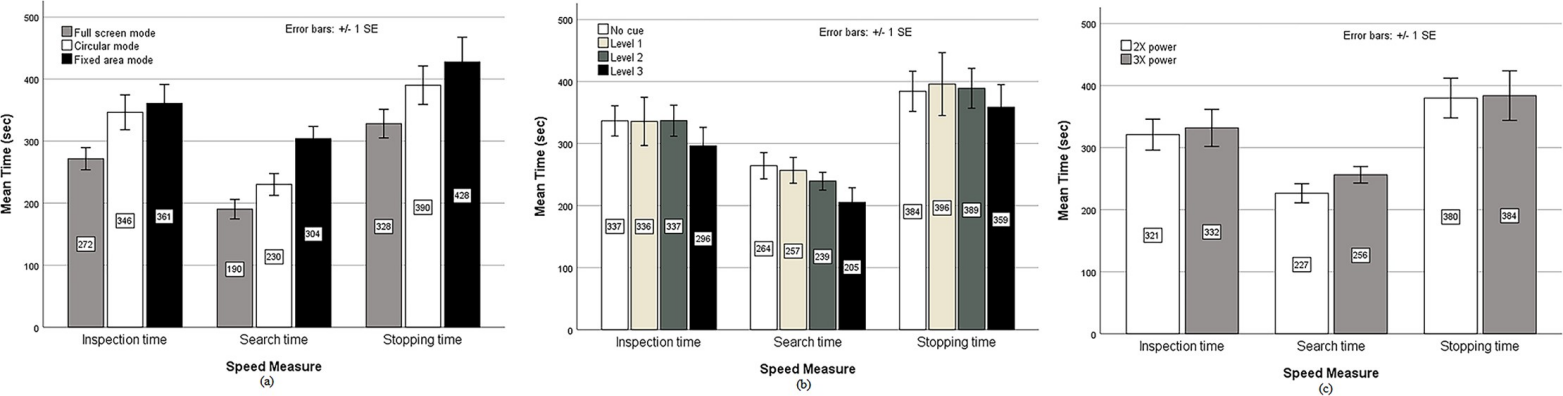
Bar charts of mean inspection times, mean search times, and mean stopping times. (A) magnification modes, (B) levels of location cues, and (C) magnification powers.

Subjects’ mean inspection times, mean search times, and mean stopping times for different magnification modes, location cue levels, and magnification powers are shown in Fig 3. Regarding the factor of magnification mode (Fig 3A), the mean *IT* for the full screen mode (272 sec) was significantly shorter than that for the circular mode (346 sec) [*t*_(27)_ = 2.491, *p* < 0.05, *d* = 0.47] and fixed-area mode (361 sec) [*t*_(27)_ = 2.672, *p* < 0.05, *d* = 0.51]. However, no significant difference in mean inspection times was noted between circular and fixed-area modes (*p* > 0.05). On mean search times, the full screen mode (190 sec) also showed the shortest time. The difference between full screen and circular modes (230 sec) was not significant (*p* > 0.05). However, the fixed-area mode (304 sec) showed significantly slower hit responses than both full screen mode [*t*_(27)_ = 4.266, *p* < 0.001, *d* = 0.81] and circular mode [*t*_(27)_ = 2.737, *p* < 0.05, *d* = 0.52]. On mean stopping times, the full screen mode (328 sec) exhibited a significantly shorter mean time than the fixed-area mode (428 sec) [*t*_(27)_ = 2.490, *p* < 0.05, *d* = 0.47], while the circular mode (390 sec) was somewhere in the middle. For the location cue factor (Fig 3B), comparable mean times were noted for the conditions of no cue, level 1, and level 2, in terms of inspection times, search times, and stopping times. Marked decreases were noted for the level 3 condition, although the time differences between the level 3 and the other three conditions were not significant (*p*’s > 0.05). For the factor of magnification power (Fig 3C), the differences in mean inspection times, mean search times, and mean stopping times between 2X and 3X magnifications were not significant (*p*’s > 0.05). To summarize, only magnification mode showed a significant effect on the speed of the inspection task.

Further analyses were done on the interaction between location cue level and magnification mode. Examinations with Student’s *t-*tests showed that for each magnification mode, the changes in mean inspection times, mean search times and mean stopping times over the four levels of location cues were not significant (*p*’s > 0.05). This suggests that the use of location cues was not effective for all the three magnification modes.

### Accuracy measure

Of the 336 target-present trials, 192 (57.14%) were hit responses and 144 (42.86%) were misses. Participants made all 336 correct rejection responses in target-absent trials and no false alarm case was found. Since the correct rejection rate and the false alarm rate were 100% and 0%, respectively, the response accuracies could be determined by the hit rate and miss rate which summed up to one. The location of the target on target-present trials was analyzed for accuracy and it was found that neither the hit nor the miss rate was affected by the location of the target. Table 2 shows the numbers and percentages of hits and misses for different magnification modes, location cue levels, and magnification powers. Analysis of response accuracies was conducted with the Chi-square tests on the number of miss responses across various factors. The results showed that the magnification mode and magnification power were significant (*p*’s < 0.05). For lowering the miss rate, the full screen mode and the lower 2X magnification should be used (*p*’s < 0.05). The location cue factor was non-significant on response accuracy in the inspection task used here (*p* > 0.05).

**Table 2:** Numbers and percentages of hit and miss responses for different magnification modes, levels of location cues, and magnification powers.

Factor	Level	Target-present trials		Total
		Number of hit responses (hit %)	Number of miss responses (miss %)	
**Magnification mode**	**Full screen**	78 (69.64%)	34 (30.36%)	112
	**Circular**	55 (49.11%)	57 (50.89%)	112
	**Fixed-area**	59 (52.68%)	53 (47.32%)	112
**Location cues**	**No cue**	45 (53.57%)	39 (46.43%)	84
	**Level 1**	47 (55.95%)	37 (44.05%)	84
	**Level 2**	47 (55.95%)	37 (44.05%)	84
	**Level 3**	53 (63.10%)	31 (36.90%)	84
**Magnification power**	**2X**	109 (64.88%)	59 (35.12%)	168
	**3X**	83 (49.40%)	85 (50.60%)	168
**Total**		192 (57.14%)	144 (42.86%)	336

### Task load index

**Fig 4:**
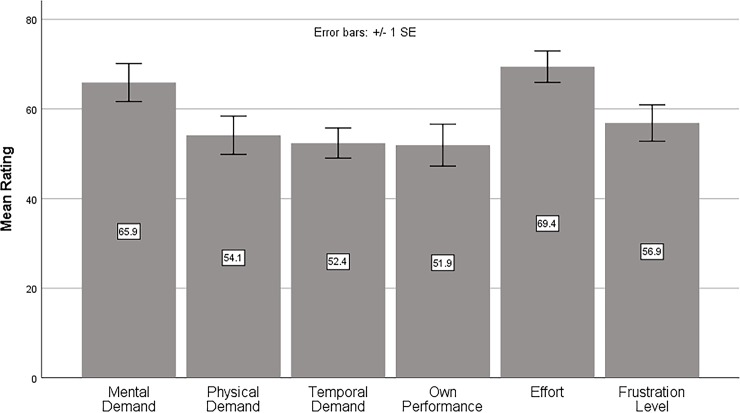
Bar chart of mean ratings of the six subscales in the Task Load Index paradigm.

In addition to the objective measures of speed and accuracy, a subjective workload evaluation was conducted on screen using the NASA Task Load Index (NASA-TLX) paradigm. The mean ratings for the six TLX subscales are shown in Fig 4. Regarding the three types of demands (0 = low; 100 = high) imposed on the participants, mental demand (65.9) was significantly higher than the physical demand (54.1) [*t*_(27)_ = 2.307, *p* < 0.05, *d* = 0.44] and temporal demand (52.4) [*t*_(27)_ = 2.722, *p* < 0.05, *d* = 0.51] whereas no significant difference was found between physical and temporal demands (*p* > 0.05). The results suggested that demand from the mental processes such as searching, decision, and memory was significantly higher than demands from physical activity and time pressure. For the other three subscales related to the interaction of participants with the inspection task, the effort score (69.4) was significantly higher than the frustration score (56.9) [*t*_(27)_ = 2.826, *p* < 0.01, *d* = 0.53] and performance score (51.9) [*t*_(27)_ = 2.775, *p* < 0.01, *d* = 0.52] whereas no significant difference was found between frustration and performance scores (*p* > 0.05). The results indicated that participants perceived the need to put greater effort into accomplishing the performance level that they perceived to be successful, while their frustration and performance satisfaction from the task were relatively low.

The mean ratings of the six TLX subscales for different main factors were further studied. For the factor of magnification mode, the full screen mode exhibited significantly lower mental demand than the circular mode [*t*_(27)_ = 2.979, *p* < 0.01, *d* = 0.56] and fixed-area mode [*t*_(27)_ = 3.029, *p* < 0.005, *d* = 0.57]. The full screen mode also imposed less physical demand on subjects than the circular mode [*t*_(27)_ = 3.178, *p* < 0.005, *d* = 0.60] and fixed-area mode [*t*_(27)_ = 3.686, *p* < 0.001, *d* = 0.70]. Besides, subjects perceived significantly better performance in the full screen mode than in the circular mode [*t*_(27)_ = 2.171, *p* < 0.05, *d* = 0.41] whereas the perceived performance in the fixed-area mode was in between the full screen and the circular modes. For the other TLX subscales of temporal demand, effort, and frustration level, the factor of magnification mode was found to be non-significant (*p*’s > 0.05).

Concerning the location cue factor, lower physical demand was found in the ‘no cue’ condition than in the ‘cued’ conditions (*p*’s < 0.05). The effect of location cues was not significant on the other five subscales. The magnification power factor was not significant on all the six subscales, indicating that subjects had comparable workload experiences when the stimulus image was magnified at either 2X or 3X.

Table 3 shows the mean TLX scores which were the weighted averages of the six subscales for different magnification interface factors. With a sample size of 168 TLX scores (28 participants × 6 test conditions), the overall mean TLX score was 61.7 (SD = 18.4). Concerning the factor of magnification mode, the results of Student’s *t*-tests on TLX measure were found to be consistent with those on objective measures of speed and accuracy. The fastest and most accurate mode of full screen magnification (54.5) imposed less workload on the participants than the circular mode (66.1) [*t*_(27)_ = 4.018, *p* < 0.001, *d* = 0.76] and fixed-area mode (64.7) [*t*_(27)_ = 3.750, *p* < 0.001, *d* = 0.71], whereas the circular and fixed modes exhibited similar workload experiences (*p* > 0.05). For the factors of location cues and magnification power, the ‘no cue’ condition (56.1) and the 2X magnification (60.4) exhibited lower mean TLX scores, although the differences between levels of location cues and between magnification powers were found to be non-significant (*p*’s > 0.05).

**Table 3:** Means and standard deviations of Task Load Index (TLX) scores for different magnification modes, levels of location cues, and magnification powers.

Factor	Level	Mean TLX scores	Standard deviation
**Magnification mode**	**Full screen**	54.5	18.6
	**Circular**	66.1	14.2
	**Fixed-area**	64.7	17.5
**Location cues**	**No cue**	56.1	16.0
	**Level 1**	65.9	14.8
	**Level 2**	60.2	14.0
	**Level 3**	64.8	15.2
**Magnification power**	**2X**	60.4	16.5
	**3X**	63.1	13.5
**Average**		61.7	18.4

### Correlations between and factor analysis on binocular near acuity, visual lobe area, search time, stopping time, miss rate, and task load index score

The Spearman correlations between binocular near acuity, visual lobe area, search time, stopping time, miss rate, and Task Load Index (TLX) score were examined. There were significant correlations between visual lobe area and search time (*r* = -0.485, *p* < 0.01), search time and stopping time (*r* = 0.421, *p* < 0.01), and miss rate and stopping time (*r* = -0.682, *p* < 0.001). Factor analysis with varimax rotation was conducted to assess the underlying relationships between the six variables. The three assumptions as tested by determinant, Kaiser-Meyer-Olkin measure, and Barlett’s test of sphericity were met [25]. Table 4 displays the loadings for the three rotated factors. The first factor which accounted for 32.79% of the variance was marked highly by the stopping time (loading = -0.921) and miss rate (loading = 0.890). The second factor which accounted for 27.66% of the variance was formed by the search time (loading = 0.828), participants’ visual lobe area (loading = -0.754), and subjective TLX score (loading = 0.585). The third factor which accounted for 18.52% was formed by participant binocular near acuity with a high loading of 0.963.

**Table 4:** Factor loadings and communalities for the rotated factors.

Variable	Factor loading			Communality
	1	2	3	
**Stopping time**	-0.921	0.202	-0.007	0.889
**Miss rate**	0.890	0.147	0.084	0.820
**Search time**	-0.278	0.828	-0.258	0.830
**Visual lobe area**	-0.082	-0.754	-0.326	0.682
**TLX score**	0.493	0.585	-0.053	0.589
**Binocular near acuity**	0.029	0.009	0.963	0.929
**Eigenvalues**	1.967	1.660	1.111	4.738
**% of variance**	32.79	27.66	18.52	78.97

## Discussion

Three interface factors of magnification mode, location cue, and magnification power were examined in this study. The results indicated a significant magnification mode effect on inspection speed, accuracy, and overall TLX score. However, the usefulness of location cues was not clearly seen as indicated by the non-significant difference in speed, accuracy, and user perception between the cued and non-cued conditions.

### Comparisons of full screen, circular, and fixed-area magnification modes

#### Search time distribution and participants’ search strategy

Previous studies have revealed that a good fit of an exponential model to the search time data indicated the likelihood of an unsystematic search strategy employed by the participants whereas a good fit of a linear model indicated the likelihood of systematic search behaviour [23, 24, 26]. In most cases, human search behaviour was found to fall somewhere between these two extremes depending on criteria such as stimulus features, presentation layouts [27], or user experience [28]. As noted in Eq 1, the exponential distribution of search times with a high coefficient of determination (*R*^*2*^) showed that in general participants employed unsystematic search strategy in this experiment. Table 5 shows the regression equations for the three magnification modes by fitting the search times separately into the linear and exponential models. Comparing the coefficients of determination between these two types of models, it seemed that participants were more likely to adopt a systematic search strategy in the full screen mode whereas they searched in an unsystematic way in the fixed-area mode. For the circular mode, the high *R*^*2*^ values obtained in both models suggested that participants might have used semi-systematic strategies, with which, for example, the viewing screen was moved from one place to another systematically over the stimulus image while the content appearing within the viewing screen was searched randomly. Based on the cumulative distributions of search times, it is believed that participants’ search strategy changed in different magnification modes.

**Table 5:** Regression equations showing the cumulative distributions of search times for the three magnification modes.

Magnification mode	Cumulative distribution of search times	*R*^*2*^ value
**Full screen**	Linear *P*(*t*_*search*_) = 0.059+0.0023*t*_*search*_	0.971
	Exponential ln[1−*P*(*t*_*search*_)] = 0.392−0.0073*t*_*search*_	0.889
**Circular**	Linear *P*(*t*_*search*_) = 0.138+0.0016*t*_*search*_	0.954
	Exponential ln[1−*P*(*t*_*search*_)] = 0.205−0.0051*t*_*search*_	0.911
**Fixed-area**	Linear *P*(*t*_*search*_) = 0.163+0.0011*t*_*search*_	0.862
	Exponential ln[1−*P*(*t*_*search*_)] = 0.231−0.0042*t*_*search*_	0.932

#### Search memory, search strategy, and search performance

A number of previous studies have shown that visual search has memory [6, 15, 29, 30]. Intuitively, the memory demand in a systematic search was much lower than that in an unsystematic search. By using a systematic search strategy, participants followed their decided scan-path (e.g. from left to right and from top to bottom) and hence there was no need for them to memorize the visited locations on the stimulus image. The problems of disorientation and lengthened inspection time or search time due to refixations were then minimised. In this study, the fixed-area mode exhibited significantly longer mean search time (304 sec) than full screen (190 sec) and circular modes (230 sec). This result gave support to the speculation of the likelihood of a systematic search strategy in the full screen mode, a semi-systematic search strategy in the circular mode, and an unsystematic search strategy in the fixed-area mode.

#### Content/contextual information and search performance

In addition to the search time, the full screen mode showed shorter mean stopping time, lower miss rate, and lower subjective workload demand perception than the circular and fixed-area modes. The screen space designs might account for the variations in participants’ performance and subjective perception for the three magnification modes. The full screen mode which displayed content information only was found to be more effective and efficient than the circular and fixed-area modes which displayed both content and contextual information. This suggested that the contextual information presented in the circular and fixed-area modes was not as useful as expected in this simulated visual inspection task. Instead, the content information was believed to be more important for ensuring good inspection performance. To summarize, the effectiveness of presenting content and contextual information on one single screen should be considered together with the nature of visual task and participants’ search behaviours in order to achieve better performance.

### Effectiveness of location cues

Unexpectedly, the aid of location cues was found to be non-significant on visual inspection performance, for all the three magnification modes. Because of the unimportance of contextual information to the inspection task, the location cues which were a kind of contextual information were found to be non-significant in the current task. It is believed that the aid of location cues will be useful when participants are asked to search certain parts of the stimulus image instead of searching over the whole image. The cues in such a case can help improve the global orientation of the image. In addition, if the visual task contained complex stimuli and an unsystematic search strategy was employed by the participants, the locations cues would be of great benefit by simplifying the memorization process or lowering the memory demand of the complex scenes that had been visited previously. Similar to the selection of magnification mode, the use of and the design for the location cues should be considered with the nature of visual tasks and participants’ search behaviours.

### Effects of magnification power and factor analysis

Regarding the interface factor of magnification power, searches with the lower 2X magnification were found to be more accurate than those with the higher 3X magnification, but these two conditions showed no significant differences in inspection speed and user perception. Results of Spearman correlations showed that the miss rate was negatively correlated with stopping time, indicating that a miss trial would most likely happen if the stopping time was short. So, the higher miss rate noted in the 3X magnification condition could possibly be due to early termination of inspection trial without sufficient search. Proper instructions regarding the standard inspection time should be given to participants before an inspection task. A simple estimate of a standard inspection time can be achieved by dividing the total area of search field by the participant visual lobe area [31]. The standard time may be increased for searches with refixations and overlapping search strategy [32]. Besides, the negative correlation of search time and visual lobe area suggested that a shorter search time (or better search performance) would be obtained from a participant with a larger visual lobe area. In contrast, no significant correlations were found between the binocular near acuity and the performance and perception measures. The results suggested that the visual lobe measurement but not the simple visual acuity test is a more useful and reliable predictor of human inspection performance.

From the results of factor analysis in Table 4, three factors were extracted which accounted for about 79% of the total variance of all six variables. The first factor formed by the miss rate and stopping time was more associated with the decision component of the visual search. In other words, the first factor related to the stopping policy or decision in the current inspection task. The second factor was formed by the search time, visual lobe area, and TLX score, and it was more related to the search component of the visual search. The first two factors explicitly indicated the importance of the two main components of search and decision in the context of visual search [33]. The last factor was the binocular near acuity of subjects. Although the acuity score measured on static visual objects was found not correlated with any of the objective performance and subjective evaluation measures in the current inspection task, it should still be tested independently in order to ensure inspectors possess minimal vision ability.

### Limitation of the study

As a pioneering study, two ordinary shapes: a circular magnified window in the circular mode and a rectangular magnified window in the fixed-area mode were chosen for examination because they were common and practical for use in the industry. Nevertheless, it is worth to have further examination of the effect of shape of magnified windows and the interaction effect of shape and magnification mode in a simple visual inspection task in future.

Similar to the shape of the magnified windows, the size of the magnified windows will also affect visual performance. In the present study, the size was determined in a way such that the stimulus objects falling within the magnified window could be seen at a single glimpse. It seems necessary to further investigate the size effect of the magnified windows and the interaction effects of size with shape and magnification mode in future research.”

Apart from the effect of shape and size of the magnified window, it is worth examining the inter-relationship between magnification mode, response criterion, and search behavior in an inspection task. Subjects’ search performance will be tested a) using different visual stimuli like searching a target in real scenes and b) manipulating the subject’s response criterion. To change subjects’ response criterion, they will be informed their performance will be measured by i) both speed and accuracy, ii) accuracy more than speed, and iii) speed more than accuracy.

The choices of target and non-target characters were basically referenced to past studies of the authors in similar visual search research [30, 34, 35]. Subjects were able to distinguish target from the non-targets while they searched. However, the stimulus type used in the present study may not be appropriate to investigate the impacts of location cues. As this is an exploratory study, further examination is needed using different visual stimulus objects.

Lastly, long inspection time and high miss rate were noted in the current visual inspection task. There is a need to redesign the simulated inspection task, such as: reducing the total search area and providing more practice before the formal tests, for better performance of the inspection task.

## Conclusion

This study showed a significant magnification mode effect and a non-significant location cue effect on visual inspection performance. It reveals that a proper selection of magnification mode is prominent to visual task performance. It is because participants’ search strategies were found varied in the full screen, circular, and fixed-area modes of magnification, and thus the inspection performance in different modes were different. In this experiment, the full screen mode was found to outperform the circular and fixed-area modes. Two reasons were suggested: the use of systematic search strategy by participants in the full screen mode and the unimportance of contextual information to the current visual inspection task.

Concerning the interface design of the limited screen space, the effectiveness of content and contextual information should be considered together with the nature of the visual task and the participants’ search behaviours. It was believed that the aid of location cues would be useful when participants employed an unsystematic search strategy and/or the task demanded high level of search memory. Beside, this study suggested the importance of standard inspection time in order to reduce the number of target misses owning to early termination of the inspection trial. Evidence was also obtained to show a positive relationship between visual lobe area and visual search performance.
